# Patient-reported prevalence of metamorphopsia and predictors of vision-related quality of life in vitreomacular traction: a prospective, multi-centre study

**DOI:** 10.1038/s41433-018-0230-0

**Published:** 2018-10-12

**Authors:** Praveen J. Patel, David H. Steel, Christoph Hirneiß, John Brazier, Abdalla Aly, Benedicte Lescrauwaet, on behalf of the MeMo Study Group

**Affiliations:** 1 NIHR Biomedical Research Centre at Moorfields Eye Hospital and UCL Institute of Ophthalmology, London, UK; 2 Sunderland Eye Infirmary, Sunderland, UK; 3 Institute of Genetic Medicine, Newcastle University, Newcastle Upon Tyne, UK; 4 Department of Ophthalmology, Ludwig-Maximilians-University, Munich, Germany; 5 School of Health and Related Research, University of Sheffield, Sheffield, UK; 6 Pharmerit International, Bethesda, MD USA; 7 Xintera bvba, Ghent, Belgium

## Abstract

**Objectives:**

To report the prevalence and severity of metamorphopsia, estimate its impact on vision-related quality of life (VRQoL) and evaluate predictors of VRQoL in patients with vitreomacular traction (VMT).

**Patients and methods:**

A prospective, cross-sectional multi-centre study in the United Kingdom of 185 patients with VMT, with or without a full thickness macular hole (FTMH). Self-reported metamorphopsia was determined using the metamorphopsia questionnaire. VRQoL was assessed using the Visual Function Questionnaire (VFQ-25). Physicians recorded clinical and ocular characteristics in both eyes including a physician assessment of metamorphopsia. ANOVA and predicted least-squares means were used to estimate the impact of metamorphopsia on VRQoL. Predictors of VRQoL were assessed using ordinary-least-squares regression adjusting for clinically important variables.

**Results:**

The prevalence of self-reported metamorphopsia was 69.7% (95% CI 62.6–76.3%) and was higher in eyes with a concomitant FTMH vs. without FTMH (85.4% vs. 64.2%). Physician assessment of metamorphopsia was 53.0% (95% CI: 45.5–60.3%). Comparing eyes with metamorphopsia vs. without metamorphopsia, the VFQ-25 composite score was lower (82.3 vs. 91.4), and mean VA (LogMAR) was worse (0.44 vs. 0.33). The largest difference in VFQ-25 scores was observed for near activities (metamorphopsia: 75.3, No metamorphopsia: 90.2). The adjusted model showed that metamorphopsia severity and age were significantly associated with lower VFQ-25 scores.

**Conclusion:**

Metamorphopsia was highly prevalent in patients with VMT and associated with significantly lower VRQoL. Physician assessment of symptoms underestimated the self-reported presence of metamorphopsia. Metamorphopsia severity acts as a predictor of impaired VRQoL, over and above decrements due to reduced vision.

## Introduction

Vitreomacular adhesion (VMA) is characterised by persistent macular attachment of the posterior vitreous within 3 mm of the fovea without an altered foveal contour and results from incomplete posterior vitreous detachment (PVD) [1]. VMA may be seen transiently as part of normal aging and occurs with higher frequency in a variety of retinal diseases including diabetic retinopathy or age-related macular degeneration (AMD) [2]. Vitreomacular traction (VMT) results when VMA is associated with alteration of foveal morphology including distortion or elevation of the foveal surface. VMT can resolve spontaneously but persistent VMT is a known risk factor for the development of a full thickness macular hole (FTMH) and epiretinal membrane (ERM) [3–5]. Patient-reported symptoms of VMT include distortion of vision (metamorphopsia) and blurred vision.

Some studies have investigated metamorphopsia and its association with vision-related quality of life (VRQoL) in retinal disorders, however none of these relate to metamorphopsia in VMT [6–13]. It could be anticipated that patients with VMT have similar signs or severity of metamorphopsia as patients with other retinal disorders who have metamorphopsia. Yet, no studies have prospectively assessed the prevalence and severity of metamorphopsia or the impact of metamorphopsia on VRQoL in patients with VMT. Although the Amsler grid allows for a simple qualitative evaluation of alterations of visual function [14], the sensitivity of the test in the early detection of metamorphopsia is low; and additionally, the test is known to have a high false-negative rate [15]. These factors may explain its limited use in current clinical practice in the UK. Further, while tools to quantify metamorphopsia such as PHP, M-charts or D-charts have been developed more recently, these instruments were not adopted in routine day-to-day clinical practice.

The aim of the present Metamorphopsia (MeMo) study was to determine the self-reported prevalence and severity of VMT-related metamorphopsia in patients presenting to eye clinics in the United Kingdom (UK), to report the impact of metamorphopsia on VRQoL, and to explore possible clinically relevant predictors of VRQoL.

## Patients and methods

### Study design and setting

The MeMo study was a prospective, observational, cross-sectional, multi-centre study conducted at NHS hospital eye clinics across the United Kingdom (UK). Ethical approval was granted by the West of Scotland Research Ethics Service (REC reference number: 14/WS/0092) and institutional R&D approval was obtained for the protocol and study-related documents. The study adhered to Good Clinical Practice and to the tenets of the Declaration of Helsinki. Informed consent was obtained from every participant prior to enrolment.

Sites were chosen to provide a representative sample of VMT patients, specifically, sites from large and small cities and a wide geographical spread across the UK. Ophthalmology clinics operating treatment protocols that included observation (watchful waiting) and/or pharmacological (ocriplasmin) or surgical (vitrectomy) vitreolysis were enroled. Participating physicians had experience with the diagnosis and treatment of VMT and were characterised by a mixed referral basis. Treating physicians continued their usual practice and patient management, no protocol-driven treatment or test was administered to preserve the observational design. Recruitment extended from July 1, 2014 until July 1, 2015.

### Participants

Inclusion criteria included a confirmed diagnosis of VMT, with or without a concomitant FTMH, within prior 6 months and ability to provide written informed consent. Consecutive patients were enroled as they presented for a routine clinic visit to address potential sources of bias. Exclusion criteria, assessed in the affected eye, included: VMT associated with an underlying macular disease (e.g., age-related macular degeneration, diabetic retinopathy; retinal vein occlusion); FTMH or epiretinal membrane (ERM) without a tractional component; traumatic MH; FTMH > 400 μm; high myopia (>8 dioptres); advanced glaucoma; prior vitrectomy or intravitreal intervention; and intraocular surgery other than vitrectomy within prior 3 months.

### Data collection

Physicians collected baseline data at the single visit for each participant using a standardised data form consisting of inclusion/exclusion criteria, patient characteristics, current medical and ocular conditions, ocular interventions, date of first symptoms, and ocular examinations. The investigator noted ocular dominance through asking the patient, using one of the recommended tests (Miles, Porta, Convergence near-point, Dolman) or alternatively the test applied in their clinical practice. In addition, the physician’s assessment of symptoms (metamorphopsia, blurred vision, curvy objects, double vision, unable to drive at night, other), ocular diagnosis and date of diagnosis were recorded. All ocular assessments were performed for the affected and fellow eye. Patients completed the metamorphopsia questionnaire and the National Eye Institute Visual Function Questionnaire, hence these were all self-reported outcomes.

### Assessment of metamorphopsia and vision-related quality of life

#### Metamorphopsia questionnaire (MeMoQ)

**Fig. 1:**
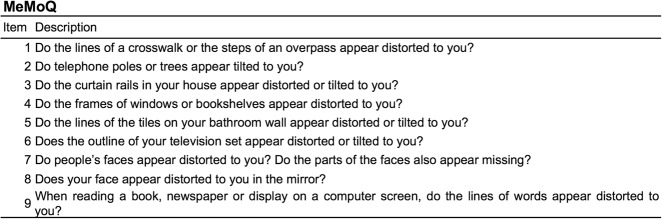
MeMoQ. The 9-item MeMoQ administered in the MeMo study. The MeMoQ analysis excluded one item (Q7) because of misfit, as concluded by the developers’ Rasch analysis. In line with the original validation study, the threshold for presence of metamorphopsia was a MeMoQ score > 0. Min–Max score: 0–3. As per the scoring algorithm, the MeMoQ score was calculated as the average of the remaining eight items using the questionnaire specific response values (“not at all” = 0 points; “a little” = 1 point; “moderately” = 2 points; “a great deal” = 3 points; items marked with “None of the above” were excluded from the scoring)

The primary outcome measure of the MeMo study was the prevalence of metamorphopsia in patients with VMT. The presence (and severity) of metamorphopsia was based on the patient’s self-reported perception of abnormal vision quality as evaluated using the MeMoQ. The metamorphopsia questionnaire, developed by Arimura et al. consists of ten items focusing on symptoms of subjective metamorphopsia in a patient’s daily life [16]. Arimura et al. previously performed a Rasch analysis to verify the questionnaire’s validity in patients with ERM, MH, AMD, and healthy controls. The questionnaire was found to be a valid assessment of patient subjective impression of metamorphopsia, and supplemented the clinical detection and quantification of metamorphopsia [16]. For the purpose of the MeMo study, we removed one item (‘Do the columns in your Japanese style rooms appear distorted or tilted to you?’) which was culturally not relevant in our European sample (Fig. 1). Consistent with the questionnaire’s scoring algorithm, the prevalence of metamorphopsia was defined as a MeMoQ score greater than zero, while severity was based on the MeMoQ score calculated as the mean score of non-missing items. Since the validation study found no difference in results between Rasch scores and total scores, our analyses were based on raw scores [16].

#### National Eye Institute Visual Function Questionnaire (NEI VFQ-25)

Patients completed the self-assessed, self-administered version of the VFQ-25 to assess VR-QoL. Each of the 25 VFQ questions is assigned to one of the 12 subscales: general health, general vision, ocular pain, near activities, distance activities, social functioning, mental health, role difficulties, dependency, driving, colour vision, and peripheral vision. Subscales scores range from 0 to 100, where 100 indicates the highest possible function or minimal subjective impairment [17]. The NEI VFQ-25 composite score was calculated as the average of the subscale scores, excluding the General Health item [18]. In line with previous clinical reports, our VFQ-25 analyses were based on raw scores.

#### Physician assessment of metamorphopsia

In addition to the primary determination of metamorphopsia prevalence (self-reported from the MeMoQ), the physician’s assessment of metamorphopsia was positive if the patient reported distorted vision and/or curvy objects in either eye.

### Study size

MeMo was a descriptive study with the primary aim of estimating the prevalence of metamorphopsia in VMT. Based on a 95% two-sided confidence interval (CI) and an assumed prevalence between 60 and 90%, a sample size of approximately 200 allows a precision of the prevalence estimate within an 8.4–13.6% range.

### Statistical methods

The prevalence of metamorphopsia assessed by the MeMoQ was computed for the overall population and for subgroups according to the presence of a FTMH. Clopper-Pearson method was performed to obtain 95% CI around prevalence estimates. Physician assessment of the presence of symptoms of metamorphopsia was summarised for the overall population and by self-reported presence of metamorphopsia. The agreement between physician and patient evaluation of metamorphopsia was calculated using the Kappa statistic [19]. Descriptive statistics for the VFQ-25 scores were generated for the overall population and by self-reported presence of metamorphopsia. *P* values assessing differences between metamorphopsia subgroups were derived from the Student’s *t*-test. Due to the exploratory nature of this study, no formal hypothesis testing was conducted. Thus, any *P*-value generated should be regarded as a descriptive, rather than inferential, statistic.

The effect of the presence of metamorphopsia on VFQ-25 was explored through a univariate (ANOVA) model. In the ANOVA model, the VFQ-25 score was the dependent variable and an indicator variable denoting presence of metamorphopsia was the independent variable. The least-square mean VFQ-25 scores were reported for patients with metamorphopsia and patients without metamorphopsia. The estimated difference between both groups were reported including 95% CI.

A multivariate ordinary least squares (OLS) regression was fit to identify the predictors of VFQ-25 scores, including metamorphopsia severity score, whether the affected eye was the best seeing eye, whether the affected eye was the dominant eye, patient age, visual acuity of best seeing eye (in LogMAR), visual acuity of the dominant eye (in LogMAR). The selection of these variables was based on clinical reasoning. Analyses were performed on patients who had non-missing data. In the scoring of questionnaire data, the rules surrounding missing items was applied for each specific questionnaire.

## Results

### Characteristics of participants

This study included a total of 185 patients enroled at 19 hospital eye clinics (Study flow diagram in Figure S1). Patients were predominantly female (67.0%), Caucasian (90.3%), with a mean age of 72.8 years (standard deviation [SD], 8.7). Almost half of the patients (46.5%) had either a concomitant or past ocular condition and 27.6% had undergone an ocular intervention prior to baseline (Table S1). Demographics and clinical characteristics were similar for patients with and without metamorphopsia, except for the occurrence of vitrectomy in the fellow eye, which was more frequently observed in patients with metamorphopsia (10/129, 7.8%) than in patients without metamorphopsia (0/56, 0%) (Table S2). The affected eye was dominant in 57.8% of patients (Table 1). The mean [SD] visual acuity (LogMAR) in the affected eye was 0.41 (0.30). Ocular characteristics of patients with metamorphopsia showed worse visual acuity (VA) (0.44; Snellen: 20/55) vs. patients without metamorphopsia (0.33; Snellen 20/43). Similarly, a concomitant FTMH was diagnosed more frequently in patients with metamorphopsia (31.8%) vs. patients without metamorphopsia (12.5%). Physician assessment of the presence of symptoms of metamorphopsia was higher in the group of patients with self-reported metamorphopsia (55.8%) vs. the group without self-reported metamorphopsia (33.9%). VMT in the fellow eye was diagnosed in 17.3% of patients; an ERM was diagnosed in 9.2% of affected eyes.

**Table 1:** Baseline demographic characteristics and ocular findings in the MeMo study population

	Overall (*n* = 185)	Metamorphopsia (*n* = 129)	No metamorphopsia (*n* = 56)	*P* value^a^
*Age (years)*				
Mean	72.8	72.8	72.8	0.981
SD	8.7	8.9	8.2	
*Gender, n (%)*				
Male	61 (33.0)	40 (31.0)	21 (37.5)	0.388
Female	124 (67.0)	89 (69.0)	35 (62.5)	
*Race, n (%)*				
Caucasian	167 (90.3)	115 (89.1)	52 (92.9)	0.141
Black	9 (4.9)	8 (6.2)	1 (1.8)	
Asian	5 (2.7)	2 (1.6)	3 (5.4)	
Other	4 (2.2)	4 (3.1)	0 (0.0)	
*Eye dominance, n (%)*				
Affected eye is dominant	107 (57.8)	76 (58.9)	31 (55.4)	0.653
*Fellow eye involvement, n (%)*				
VMT present in fellow eye	32 (17.3)	21 (16.3)	11 (19.6)	0.578
*Physician assessment of metamorphopsia*				
In affected eye	91 (49.2)	72 (55.8)	19 (33.9)	0.006
In fellow eye	21 (11.4)	16 (12.4)	5 (8.9)	0.494
*Retinal exam findings in affected eye* ^b^ *, n (%)*				
Clinically evident VMT	149 (80.5)	101 (78.3)	48 (85.7)	0.242
FTMH	42 (22.7)	35 (27.1)	7 (12.5)	0.029
*Additional OCT findings in affected eye* ^b^ *, n (%)*				
FTMH present	46 (24.9)	39 (30.2)	7 (12.5)	0.010
ERM present	18 (9.7)	13 (10.1)	5 (8.9)	0.809
*Physician assessment of ocular diagnosis in affected eye, n (%)*				
FTMH	48 (25.9)	41 (31.8)	7(12.5)	0.006
ERM	17 (9.2)	13 (10.1)	4 (7.1)	0.526
*Physician assessment of ocular diagnosis in any eye, n (%)*				
FTMH	55 (29.7)	47 (36.4)	8 (14.3)	0.002
ERM	25 (13.5)	20 (15.5)	5 (8.9)	0.229
*Visual acuity (LogMAR)* ^c^				
Affected eye, *n*	183	128	55	0.038
Mean (SD)	0.41 (0.30)	0.44 (0.32)	0.33 (0.25)	
Fellow eye, *n*	176	124	52	0.371
Mean (SD)	0.28 (0.39)	0.30 (0.38)	0.24 (0.41)	
*Size of adhesion (microns)* ^c^				
*n*	165	112	53	0.095
Mean (SD)	440.3 (477.5)	397.6 (408.1)	530.6 (592.6)	
*Size of macular hole (microns)* ^c^				
*n*	43	36	7	0.572
Mean (SD)	236.6 (98.9)	240.4 (100.5)	217.0 (94.8)	
*Central/Macular subfield thickness (microns)* ^c^				
*n*	150	104	46	0.296
Mean (SD)	334.5 (84.6)	339.3 (79.3)	323.6 (95.5)	

Metamorphopsia, where presence of metamorphopsia was defined as a metamorphopsia questionnaire (MeMoQ) score > 0; No Metamorphopsia, where absence of metamorphopsia was defined as a MeMoQ score = 0

*SD* standard deviation, *LogMAR* logarithm of the minimum angle of resolution, *VMT* vitreomacular traction, *FTMH* full-thickness macular hole, *ERM* epiretinal membrane

^a^*P* values assessing difference between metamorphopsia subgroups were derived from the chi-square and Student’s *t*-test for categorical and continuous variables, respectively.

^b^Categories not mutually exclusive; percentages may not add to 100%

^c^Includes patients with measured visual acuity or OCT. Patients not included in this calculation had Unknown/Not Measured indicated on the CRF

### Self-reported prevalence and severity of metamorphopsia

The prevalence and severity of metamorphopsia was based on the patient’s subjective perception of metamorphopsia as evaluated using the MeMoQ questionnaire, a self-assessed and self-administered questionnaire. The overall self-reported prevalence of metamorphopsia was 69.7% (95% CI 62.6, 76.3%) and higher among patients with a concomitant FTMH (85.4%; 95% CI 72.2, 93.9%) vs. patients with no FTMH (64.2%; 95% CI 55.6, 72.2%; Figure S2). When a FTMH was present the severity was higher (0.66; SD: 0.63) vs. patients with no FTMH (0.36; SD: 0.52; Figure S3).

### Agreement between self-reported (MeMoQ) and physician assessed presence of metamorphopsia

The patient’s perception of metamorphopsia was based on the results of the MeMoQ, and self-reported (Fig. 1). The physician assessment of metamorphopsia was based on the physician asking the patient if he/she experienced any visual symptoms (metamorphopsia, blurred vision, curvy objects, double vision, unable to drive at night, other), in the affected or fellow eye (Figure S4 symptom-based questionnaire). The presence of metamorphopsia according to the physician’s assessment of symptoms was 53.0% (95% CI 45.5, 60.3%) and higher in patients with (59.7%; 95% CI 50.7, 68.2%) vs. patients without metamorphopsia (37.5%; 95% CI 24.9, 51.5%; Figure S5).

Consistent with the MeMo study objective, the prevalence of metamorphopsia (69.7%) was primarily based on self-reported perception of subjective symptoms of metamorphopsia. Furthermore, physicians assessed the presence of metamorphopsia symptoms, denoted as distorted vision and/or curvy objects in any eye, which was lower (53.0%). The agreement between self-reported (MeMoQ) and physician assessment (symptom-based) of the presence/absence of metamorphopsia was concordant in 112 patients (60.5%). (In 77 patients the self-reported assessment on the presence of metamorphopsia agreed with the physician’s and in 35 patients the self-reported assessment on the absence of metamorphopsia agreed with the physician’s assessment). The assessment of metamorphopsia status was discordant in 73 (52 + 21) patients (39.5%). The Kappa statistic measuring the difference between observed and expected agreement (by chance alone) was 0.192 (95% CI 0.058–0.326) indicating a slight agreement (Table 2).

**Table 2:** Agreement between patient and physician assessment of metamorphopsia

		Self-reported assessment (MeMoQ-based)		
		Metamorphopsia	No metamorphopsia	Total
Physician assessment (symptom-based)	Metamorphopsia	**77**	*21*	98
	No Metamorphopsia	*52*	**35**	87
		129	56	185

Agreement between self-reported and physician assessment of metamorphopsia

Physician assessment: Assessment of metamorphopsia symptoms denoted as presence of distorted vision and/or curvy objects, in any eye; Patient assessment: Self-reported presence of metamorphopsia, defined as a MeMoQ score > zero (*N* = 129). Values in bold represent agreement (77 + 35) between patient and physician assessment. Values in italic represent disagreement (52 + 21) between patient and physician assessment

### Vision-related quality of life

**Fig. 2:**
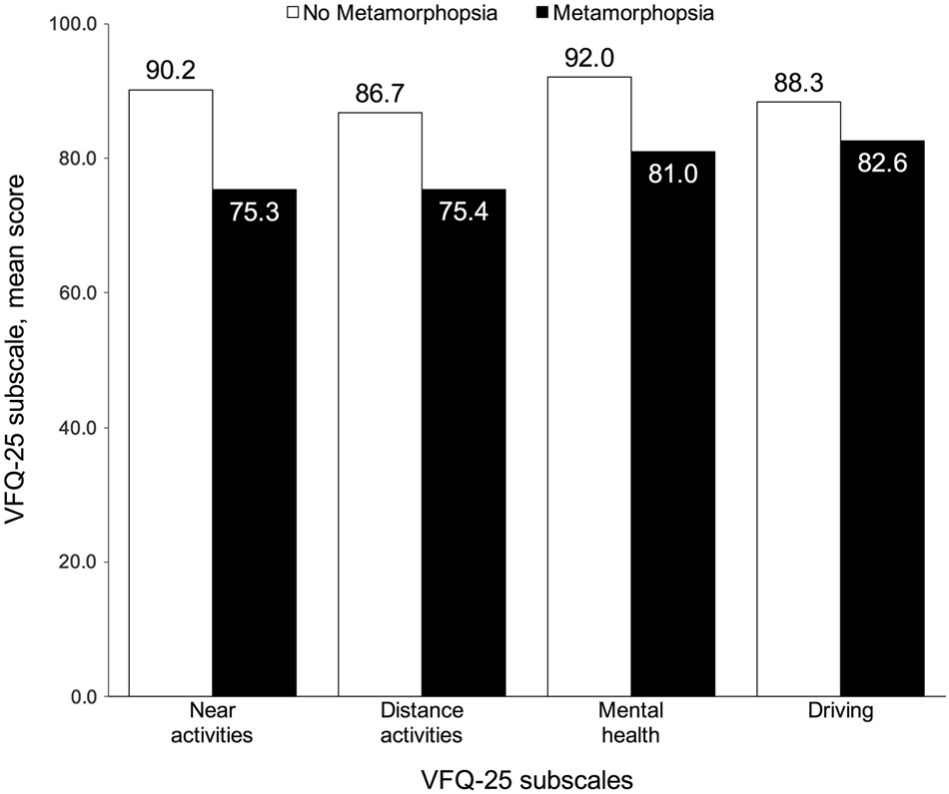
Main VFQ-25 subscales by presence of metamorphopsia Descriptive statistics for the main VFQ-25 subscales were generated for the study population by self-reported presence of metamorphopsia. *P* values assessing differences between metamorphopsia subgroups were derived from the Student’s *t*-test. Mean scores were noticeably lower (indicating poorer VRQoL) in patients with vs. patients without metamorphopsia for the main VFQ-25 subscales (*P* < .05)

Vision-related quality of life was self-assessed by the patient and based on results of the VFQ-25 questionnaire (self-administered version). VRQoL, as measured by the VFQ-25 composite score was 85.1 points in the overall population and was markedly lower in patients with metamorphopsia (82.3) vs. patients without metamorphopsia (91.4; Figure S6). For individual domains, the largest difference in mean scores between both subgroups was observed for near activities (metamorphopsia: 75.3, No metamorphopsia: 90.2, difference: 14.9). Differences for distance activities and mental health were 11.3 (75.4 vs. 86.7) and 11.0 points (81.0 vs. 92.0), respectively (Fig. 2). The smallest difference in mean score (4.8 points) was observed for colour vision (95.2 vs. 100; Figure S6). Overall, mean scores were noticeably lower (indicating poorer VRQoL) in patients with vs. patients without metamorphopsia for all domains (*P* < .05), except for the driving subscale and general health item VFQ-25 (Figure S6).

### Impact of metamorphopsia on vision‐related quality of life

Based on the univariate model that examined the impact of the presence of metamorphopsia on VFQ-25, the mere presence of metamorphopsia was associated with a significantly lower VFQ-25 score. The mean VFQ-25 composite score for patients with metamorphopsia was 82.3 (95% CI 79.8, 84.9) vs. 91.4 (95% CI 87.5, 95.3) for patients without metamorphopsia. Therefore, the disutility associated with the presence of metamorphopsia was −9.1 (95% CI −13.7, −4.4; *P* < .001).

Subgroup analyses further investigated the presence of concomitant FTMH or ERM on these quality of life findings. More specifically, 121 patients had an isolated VMT (no FTMH, no ERM) in the affected eye, while 64 patients had a concomitant FTMH or ERM. Results indicate that quality of life (as measured by the VFQ-25 composite score) was lower in VMT patients when metamorphopsia was present (82.90, *n* = 76) compared to patients without metamorphopsia (90.99, *n* = 45). This difference of 8.09 points was significant (*P* = 0.0015). Similar findings were observed in patients with a concomitant FTMH or ERM: quality of life in patients with metamorphopsia was 81.54 (*n* = 53) compared with 93.12 (*n* = 11) in patients without metamorphopsia. This difference of 11.58 points was significant (*P* = 0.046). A similar analysis using any-eye level data confirmed that the presence of metamorphopsia significantly impacts vision-related QoL regardless of the ocular diagnosis (Table S3).

### Predictors of vision‐related quality of life

The OLS regression model estimated the effect of the different covariates on VFQ-25 composite score (Table 3). The variables that were significantly associated with lower VFQ-25 scores were severity of metamorphopsia (*P* < 0.001) and patient age (*P* < 0.05). Each unit increase in metamorphopsia severity score was associated with a 14.5-point reduction in the VFQ-25 composite score. Each additional 10 years in patient’s age was associated with 3-point reduction in VFQ-25. VA of the best seeing eye was marginally predictive of VFQ-25 (*P* = 0.086).

**Table 3:** Multivariate regressions of independent predictors for VR-QoL

Independent variable	Parameter Estimate	Standard error	Lower 95% CI	Upper 95% CI	*P* value
Intercept	114.31	9.18	96.19	132.44	<0.001
Affected eye is best seeing eye^a^	−4.82	2.78	−10.31	0.68	0.086
Affected eye is dominant eye^a^	2.65	2.44	−2.17	7.46	0.279
Patient age^b^	−0.3	0.12	−0.55	−0.06	0.016
Visual acuity of best seeing eye (in LogMAR) ^b^	−2.19	3.91	−9.9	5.56	0.580
Visual acuity of dominant eye (in LogMAR) ^b^	−4.22	4.62	−13.36	4.91	0.362
Metamorphopsia severity score^b^	−14.55	1.95	−18.4	−10.69	<0.001

A parsimonious model refers to the simplest plausible model with the fewest possible number of variables. No automatic variable selection methods were employed since explorative models include many variables that are highly correlated

^a^Value: yes vs. no

^b^Value: continuous

## Discussion

In patients with VMT, metamorphopsia is considered one of the cardinal symptoms which impairs a patient in the ability to perform activities such as reading, face recognition, cooking, watching television and driving. This study shows that 69.7% of patients with VMT attending NHS hospital eye clinics in the UK reported the presence of metamorphopsia. The prevalence was notably higher among VMT patients with a concomitant FTMH (85.4%) compared to patients with no FTMH (64.2%). Patients who self-reported metamorphopsia had a noticeably lower VRQoL compared to patients with no metamorphopsia (a 9.1-point decrease on the VFQ-25 composite score). Moreover, the independent effect of the severity of metamorphopsia, over and above VRQoL decrements due to reduced VA, is an important finding indicating that despite controlling for vision, metamorphopsia impacts the quality of vision.

The present literature reveals a paucity of data on the prevalence and severity of metamorphopsia self-reported by patients with VMT. Indeed, there are no prospective reports from multi-centre settings relating to metamorphopsia in VMT in clinical practice. However, the recent interest in pharmacotherapy for the treatment of VMT generated data relating to symptoms and VRQoL in VMT from clinical trials. The OASIS study [20] was a multi-centre clinical trial of ocriplasmin for the treatment of VMT and the prevalence of metamorphopsia (detected using Amsler grid testing) was 70.2% in patients with VMT only, compared with 92.1% in VMT patients with a concomitant FTMH [21]. The MeMo study was an observational study of routine practice and metamorphopsia testing was not routinely measured in clinical practice in a measurable objective way. It is interesting however how similar the self-reported prevalence of metamorphopsia was in the MeMo study, using the MeMo questionnaire, as compared to Amsler testing in the OASIS study. The numerically higher prevalence reported in the OASIS study compared to the MeMo study may result from differences in measurement tools (Amsler grid vs. self-reported MeMoQ), settings (clinical trial vs. usual clinical practice) and differences in patient characteristics.

One major finding in the MeMo study is the discordance in self-reported presence of metamorphopsia using the MeMoQ versus patient-reported symptoms on questioning by the ophthalmologist. When metamorphopsia was diagnosed based on the ophthalmologist’s assessment of symptoms of distorted vision and/or curvy objects in any eye, rather than patient self-assessment, the prevalence was lower (53.0%). Both the presence of metamorphopsia and VRQoL were self-reported and reflect a patient-level (not eye-specific) assessment. Similarly, physicians assessed symptoms in affected and fellow eye. The analysis of agreement between physician and self-reported assessments used data for any eye, hence, the discordance cannot be attributed to a mono vs. binocular assessment. Several factors may underlie this disparity including misinterpretation of the questions posed by the ophthalmologist, differences between physicians’ questions and the MeMoQ, or patients feeling unsure whether to disclose the full extent of symptoms. Disparities between patients and physicians in reporting or describing symptoms have been reported previously, including retinal disorders [22, 23].

Our results show impaired VRQoL using the VFQ-25 in patients with recently diagnosed VMT. The MIVI-TRUST clinical programme reported a mean baseline VFQ-25 composite score of 82 points for placebo-treated and 77.1 for ocriplasmin-treated patients [24]. This VMT population was further characterised by a mean baseline BCVA of 64.3 letters, a markedly high prevalence of ERM (38.7%), and a presence of a concomitant FTMH in 23.5% of patients compared to a mean VA of 64.5 letters (0.41 LogMAR), 13.5 % of patients with ERM and 25.9% with FTMH in the MeMo study [25]. Despite these differences between the MIVI-TRUST and the MeMo population, our results show that the VFQ-25 composite score observed in the MeMo group is reasonably consistent with the VFQ-25 findings by Stalmans et al.

After adjusting for baseline VA, age, and other clinically important variables, the severity of metamorphopsia was the most predictive of impaired VRQoL. These results may under-estimate the full impact of metamorphopsia since the multivariate analysis controls for VA and other variables that may in themselves be affected by metamorphopsia. Nevertheless, the results underscore the importance of metamorphopsia as a predictor for VRQoL in patients with VMT. Surprisingly, the involvement of the dominant eye did not appear to affect VRQoL. This could be because the measurement of dominance is influenced by the pathology (i.e., less reliable if one eye is affected) or due to the crude method of assessment of eye dominance used in clinical practice.

A number of previous studies have assessed the severity of metamorphopsia in other retinal disorders, using Amsler grid, M-CHARTS and/or PHP, and found that changes in the severity of metamorphopsia was an important factor of changes in VRQoL [6–8, 10–12]. It could be postulated that the impact of metamorphopsia on VRQoL is similar regardless of the cause of the retinal disorder, though previous studies have not assessed metamorphopsia and its association with VRQoL in patients with VMT.

In the MeMo study, a diagnosis of VMT was reported in 17% of fellow eyes (bilateral affection), and physicians assessed the presence of symptoms of metamorphopsia in 11% of fellow eyes. Impairment in VRQoL could partially be attributed to VMT or macular pathology in the fellow eye, however, based on the results of the multivariate regression analysis adjusting for covariates such as fellow eye involvement did not significantly affect the impact of metamorphopsia on VFQ-25 composite scores. In addition, subgroup analyses confirmed that the presence of metamorphopsia significantly impacted vision-related quality of life both in patients with isolated VMT as well as in patients with a concomitant FTMH or ERM.

The MeMo study is the first prospective study using the questionnaire developed by Arimura et al. to assess metamorphopsia based on self-reported perception of abnormal vision quality.

This study has several strengths, including the use of a prospective, multi-centre study design with large sample size and the collection of rich phenotypic data including information from SD-OCT imaging as well as patient-centred reporting of metamorphopsia and VRQoL. The limitations include the lack of use of a wider range of methods to quantify the severity of metamorphopsia due to the time limitations on patients and clinicians in real-world clinical settings (absence of a validated instrument for the clinical evaluation of metamorphopsia at the time of study design). Indeed, at a feasibility study at the time of the MeMo study design confirmed unanimously that diagnostic tools to detect metamorphopsia in the UK NHS eye clinics, such as Amsler grid, were not used in clinical practice because of their inability to quantify the degree of metamorphopsia. In addition, no validated tool was available or adopted in UK clinical practice, meaning the introduction of such objective measurement instrument would have altered real life (observational) practice. Additional research to detect and assess metamorphopsia with validated tools is warranted to investigate the correlation between the patient’s perception of the severity of metamorphopsia and an objective quantification. Although reading vision is a key component in the assessment of metamorphopsia, an important limitation of our study was the absence of this assessment. Indeed, near vision function was affected in close to half of the patients. Further research to include an assessment of full visual function performance such as reading acuity or contrast sensitivity is warranted. Finally, the MeMo study population was restricted to patients with a FTMH diameter smaller than or equal to 400 µm, to be consistent with the population studied in the ocriplasmin clinical trials. Although this may restrict generalisability of the results, it allows better comparability with the existing evidence on VFQ-25 outcomes in VMT patients eligible for pharmacological treatment.

In summary, the MeMo Study is a large prospective patient-centred study reporting the prevalence and severity of self-reported metamorphopsia and its independent effect on VRQoL in patients recently diagnosed with VMT attending hospital eye clinics in the UK. The results show that metamorphopsia is a highly prevalent symptom, particularly in those with a concomitant FTMH, and impairs VRQoL independent of the presence of a FTMH and reduced VA. Given the importance of metamorphopsia as a symptom in patients with VMT, it is important to consider the impact of new and existing treatments on this disabling symptom. Further research supporting a full psychometric evaluation of the questionnaire in populations with retinal disorders, including VMT is warranted.

### Summary

#### What was known before

- A small number of studies have reported the prevalence of metamorphopsia (distorted vision) and its association with impaired vision-related quality of life (VRQoL) in retinal disorders.
- None of these relate to metamorphopsia in patients with vitreomacular traction (VMT).

#### What this study adds

- Metamorphopsia is a common symptom of abnormal vision in patients recently diagnosed with VMT, and is a predictor of impaired quality of life, over and above quality of life decrements due to reduced visual acuity. Increasing severity of metamorphopsia and age were associated with a poorer vision related quality of life, independently of visual acuity.
- Metamorphopsia was reported more frequently when using a metamorphopsia-specific patient questionnaire as compared to symptom-based assessment by the ophthalmologist.
- Given the importance of metamorphopsia as a symptom in patients with VMT, the use of a patient-reported questionnaire to detect symptoms of metamorphopsia may be considered in clinical practice.

## Electronic supplementary material

MeMo supplementary figures and tables
