# Supplementary material for: Patient-reported prevalence of metamorphopsia and predictors of vision-related quality of life in vitreomacular traction: a prospective, multi-centre study
Source: Eye (Lond). 2018 Oct 12;33(3):435–44. doi: 10.1038/s41433-018-0230-0 (PMC6460702; doi:10.1038/s41433-018-0230-0)
Supplement: Supplementary file 1 — MeMo supplementary figures and tables [file 41433_2018_230_MOESM1_ESM.docx]

**Figure S1. MeMo study flow diagram**

The MeMo study analysis population consisted of 185 patients enrolled between July 2014 and July 2015 at 19 hospital eye clinics.

**Figure S2. Prevalence of Metamorphopsia in the MeMo study population.**

Prevalence of metamorphopsia was defined as a MeMoQ score greater than zero. Overall: total MeMo study population (N=185); No MH: patients without concomitant macular hole (N=137); MH: patients with concomitant macular hole (N=48). P values assessing differences between macular hole subgroups were derived from the chi-square test.

**Figure S3. Severity of metamorphopsia in the MeMo study population**

Severity of metamorphopsia was based on the MeMoQ score calculated as the mean score of non-missing items using the questionnaire specific response values (“not at all” = 0 points; “a little” = 1 point; “moderately” = 2 points; “a great deal” = 3 points; items marked with “None of the above” were excluded from the scoring) (Min-Max score: 0 = no MM to 3 = severe MM); Overall: total MeMo study population (N=185); Metamorphopsia present, where presence of metamorphopsia was defined as a MeMoQ score >0 (N=129); No MH: patients without concomitant MH (N=137); MH: patients with concomitant MH (N=48). P values assessing differences between macular hole subgroups were derived from the Student’s t-test.

**
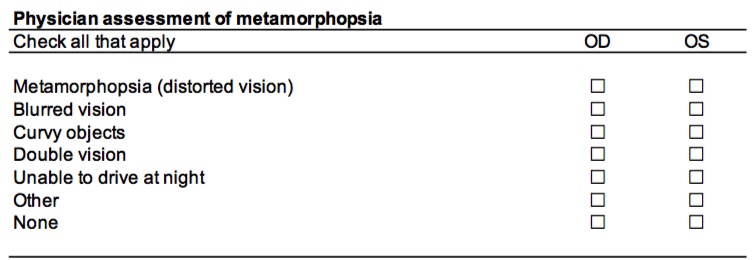
**

**Figure S4. Physician symptom-based assessment of metamorphopsia**

Physician assessment of metamorphopsia was symptom-based and denoted as presence of distorted vision and/or curvy objects, in any eye.

**Figure S5. Physician assessment of metamorphopsia symptoms**

Physician assessment of metamorphopsia was symptom-based and denoted as presence of distorted vision and/or curvy objects, in any eye; Metamorphopsia present, where self-reported presence of metamorphopsia was defined as a MeMoQ score greater than zero (N=129); No metamorphopsia: metamorphopsia absent, where absence of metamorphopsia was defined as a MeMoQ score equal to zero (N=56). P values assessing differences between metamorphopsia subgroups were derived from the chi-square test.

**Figure S6. Vision-related quality of life: NEI VFQ-25 composite and subscale scores by presence of metamorphopsia**

P values assessing differences between metamorphopsia subgroups were derived from the Student’s t-test. P<0.05 for patients with metamorphopsia vs. patients without metamorphopsia for all VFQ-25 subscales, except for the driving subscale and general health item.

**Table S1. Ocular conditions and interventions prior to baseline**

|  |  | Any eye†  (N=185) | Study eye  (N=185) | Fellow eye  (N=185) |
| --- | --- | --- | --- | --- |
| ***Current ocular condition, n (%)*** | None | 99 (53.5) | 117 (63.2) | 112 (60.5) |
|  | Cataract | 42 (22.7) | 38 (20.5) | 35 (18.9) |
|  | Glaucoma or ocular hypertension | 5 (2.7) | 4 (2.2) | 4 (2.2) |
|  | Retinal disorder | 29 (15.7) | 11 (5.9) | 25 (13.5) |
|  | Other | 34 (18.4) | 26 (14.1) | 22 (11.9) |
| ***Previous***  ***ocular***  ***interventions,***  ***n (%)*** | None | 134 (72.4) | 159 (85.9) | 137 (74.1) |
|  | Vitrectomy | 10 ( 5.4) | 0 ( 0.0) | 10 ( 5.4) |
|  | Cataract surgery | 34 (18.4) | 23 (12.4) | 31 (16.8) |
|  | Intravitreal injection | 12 ( 6.5) | 0 ( 0.0) | 12 ( 6.5) |
|  | Laser | 1 ( 0.5) | 0 ( 0.0) | 1 ( 0.5) |
|  | Glaucoma surgery | 1 ( 0.5) | 0 ( 0.0) | 1 ( 0.5) |
|  | Other | 9 ( 4.9) | 6 ( 3.2) | 7 ( 3.8) |
| †Assessment at patient level | | | | |

**Table S2. Baseline demographic and clinical characteristics of the MeMo study population**

|  | | Overall (N=185) | Metamorphopsia (N=129) | No Metamorphopsia (N=56) | P value |
| --- | --- | --- | --- | --- | --- |
| ***Age (yrs)*** |  | | | | |
| Mean  SD | | 72.8  8.7 | 72.8  8.9 | 72.8  8.2 | 0.981 |
| ***Gender, n (%)*** | | | | | |
| Male | | 61 (33.0) | 40 (31.0) | 21 (37.5) | 0.388 |
| Female | | 124 (67.0) | 89 (69.0) | 35 (62.5) |  |
| ***Race, n (%)*** | | | | | |
| Caucasian | | 167 (90.3) | 115 (89.1) | 52 (92.9) | 0.141 |
| Black | | 9 (4.9) | 8 (6.2) | 1 (1.8) |  |
| Asian | | 5 (2.7) | 2 (1.6) | 3 (5.4) |  |
| Other | | 4 (2.2) | 4 (3.1) | 0 (0.0) |  |
| ***Current medical condition^1^, n (%)*** | | | | | |
| None | | 58 (31.4) | 42 (32.6) | 16 (28.6) | 0.591 |
| Hypertension | | 64 (34.6) | 44 (34.1) | 20 (35.7) | 0.833 |
| Diabetes | | 33 (17.8) | 23 (17.8) | 10 (17.9) | 0.996 |
| Hypercholesterolemia | | 34 (18.4) | 26 (20.2) | 8 (14.3) | 0.344 |
| Depression | | 11 (5.9) | 9 (7.0) | 2 (3.6) | 0.368 |
| CVD (angina, stroke, MI) | | 25 (13.5) | 18 (14.0) | 7 (12.5) | 0.790 |
| Other | | 79 (42.7) | 57 (44.2) | 22 (39.3) | 0.536 |
| ***Current ocular condition in any eye^1^, n (%)*** | | | | | |
| None | | 99 (53.5) | 69 (53.5) | 30 (53.6) | 0.992 |
| Cataract | | 42 (22.7) | 28 (21.7) | 14 (25.0) | 0.623 |
| Glaucoma or ocular hypertension | | 5 (2.7) | 3 (2.3) | 2 (3.6) | 0.631 |
| Retinal disorder | | 29 (15.7) | 21 (16.3) | 8 (14.3) | 0.732 |
| Other | | 34 (18.4) | 25 (19.4) | 9 (16.1) | 0.593 |
| ***Ocular interventions in any eye^1^, n (%)*** | | | | |  |
| None | | 134 (72.4) | 89 (69.0) | 45 (80.4) | 0.112 |
| Vitrectomy | | 10 ( 5.4) | 10 (7.8) | 0 ( 0.0) | 0.032 |
| Cataract surgery | | 34 (18.4) | 27 (20.9) | 7 (12.5) | 0.174 |
| Intravitreal injection | | 12 ( 6.5) | 8 ( 6.2) | 4 ( 7.1) | 0.811 |
| Laser | | 1 ( 0.5) | 0 ( 0.0) | 1 ( 1.8) | 0.128 |
| Glaucoma surgery | | 1 ( 0.5) | 0 ( 0.0) | 1 ( 1.8) | 0.128 |
| Other | | 9 ( 4.9) | 7 ( 5.4) | 2 ( 3.6) | 0.590 |
| Patients with metamorphopsia were identified as those with a MeMoQ score > 0, while patients without metamorphopsia were identified as those with a MeMoQ score equal to 0; SD: standard deviation; CVD: Cardiovascular disease; MI: Myocardial infarction. ^1^Categories not mutually exclusive; percentages may not add to 100%. P values assessing difference between metamorphopsia subgroups were derived from the chi-square and Student’s t-test for categorical and continuous variables, respectively. | | | | | |

**Table S3. Subgroup analyses: Impact of metamorphopsia on vision-related quality of life by ocular diagnosis (isolated VMT vs. concomitant FTMH or ERM)**

| ***AFFECTED EYE*** | | | | | | |
| --- | --- | --- | --- | --- | --- | --- |
| **Isolated VMT** | n | Mean | SE | SD | 95% CI | |
| MM absent | 45 | 90.99 | 1.34 | 9.00 | 88.29 | 93.70 |
| MM present | 76 | 82.90 | 1.75 | 15.23 | 79.42 | 86.38 |
| Total | 121 | 85.91 | 1.25 | 13.79 | 83.43 | 88.39 |
| diff |  | 8.09 | 2.50 |  | 3.15 | 13.04 |
| diff = mean (no MM) - mean (MM) | | | |  | t | 3.2421 |
| Pr(\|T\| > \|t\|) = 0.0015 |  |  |  |  | df | 119 |
| **FTMH or ERM** | n | Mean | SE | SD | 95% CI | |
| MM absent | 11 | 93.12 | 2.02 | 6.71 | 88.61 | 97.62 |
| MM present | 53 | 81.54 | 2.54 | 18.51 | 76.44 | 86.64 |
| Total | 64 | 83.53 | 2.20 | 17.58 | 79.13 | 87.92 |
| diff |  | 11.58 | 5.69 |  | 0.21 | 22.95 |
| Diff = mean (no MM) - mean (MM) | | | | | t | 2.0365 |
| Pr(\|T\| > \|t\|) =0.046 |  |  |  |  | df | 62 |
| ***ANY EYE*** | | | | | | |
| **Isolated VMT** | n | Mean | SE | SD | 95% CI | |
| MM absent | 43 | 91.10 | 1.39 | 9.09 | 88.30 | 93.90 |
| MM present | 67 | 82.73 | 1.90 | 15.55 | 78.94 | 86.53 |
| Total | 110 | 86.00 | 1.33 | 13.97 | 83.36 | 88.64 |
| diff |  | 8.37 | 2.62 |  | 3.17 | 13.56 |
| Diff = mean (no MM) - mean (MM) | | | |  | t | 3.1914 |
| Pr(\|T\| > \|t\|) = 0.0019 |  |  |  |  | df | 108 |
| **FTMH or ERM** | n | Mean | SE | SD | 95% CI | |
| MM absent | 13 | 92.44 | 1.90 | 6.84 | 88.30 | 96.57 |
| MM present | 62 | 81.91 | 2.26 | 17.78 | 77.40 | 86.43 |
| Total | 75 | 83.74 | 1.95 | 16.86 | 79.86 | 87.62 |
| diff |  | 10.52 | 5.03 |  | 0.50 | 20.54 |
| Diff = mean (no MM) - mean (MM) | | | | | t | 2.0926 |
| Pr(\|T\| > \|t\|) =0.0399 |  |  |  |  | df | 73 |
